# Trends in extended spectrum beta-lactamase (ESBL) producing Enterobacteriaceae and ESBL genes in a Dutch teaching hospital, measured in 5 yearly point prevalence surveys (2010-2014)

**DOI:** 10.1186/2047-2994-4-S1-P123

**Published:** 2015-06-16

**Authors:** I Willemsen, S Oome, C Verhulst, A Pettersson, K Verduin, J Kluytmans

**Affiliations:** 1 Laboratory of Microbiology and Infection control, Amphia Hospital, Breda, Netherlands; 2 Department for Medical Microbiology and Infection control, VU University Medical Center, Amsterdam, Netherlands; 3 Julius Center for Health Sciences and Primary Care, UMC Utrecht, Utrecht, Netherlands

## Introduction

For the execution of a good infection control policy we depend on information about the local endemic level of resistant microorganisms and resistance genes.

## Objectives

This paper describes the trends in prevalence of ESBL producing Enterobacteriaceae (ESBL-E) and ESBL genes, measured in five consecutive yearly Point Prevalence Surveys (PPS), in a Dutch teaching hospital.

## Methods

On the day of the survey all patient present in the hospital and day-care clinic (including patients on dialyses), were screened for rectal ESBL-E carriage. Rectal swabs (Eswab, Copan, Italy) were taken and cultured using an enrichment broth, containing cefotaxime (0.25 mg/L) and vancomycin (8 mg/L) (TSB-VC) and a selective agar plate (EbSA, Alpha-Omega, Netherlands). Both phenotypical and genotypical methods were used to detect the production of ESBL and presence of ESBL-genes. Isolates containing an identical ESBL gene, from patients that were admitted on the same ward, were selected for Amplified Fragment Length Polymorphism typing to identify clonal relatedness.

## Results

Out of 2,695 patients who were screened and evaluable, 135 (5.0%) were positive for ESBL-E. *E. coli* was most frequently found (112/145), followed by *K. pneumoniae* (9/145), and *E.cloacae* (7/145). The ESBL-E prevalence was stable over the years. In all PPSs CTX-M ESBLs were the most prevalent ESBL type. Over the years, a decrease in CTX-M-1-1 like ESBL genes was observed, starting with a proportion of 44% in 2010, 34% in 2011, 22% in 2012, 24% in 2013 to 25% in 2014 (*p*=0.026). Overall 5.2% of all ESBL-E were acquired by nosocomial transmission based on epidemiological linkage and molecular typing of the strains.

## Conclusion

During this 5-year period the prevalence of rectal ESBL-E carriage was stable and only a minority was caused by nosocomial transmission. A relative decrease of CTX-M-1-1 like ESBL genes was observed. As this is the most prevalent ESBL gene in poultry, this decrease might be related to the strong (>60%) decrease in the use of antibiotics in poultry in our country in the same period.

## Disclosure of interest

None declared.

